# Dynamic Changes in Postprandial Plasma Free Amino Acid Levels of the Hepatic Portal, Hepatic, and Jugular Veins in the Healthy Pre‐Ruminant Calves

**DOI:** 10.1111/asj.70058

**Published:** 2025-04-01

**Authors:** HueyShy Chee, Atsushi Kimura, Aiko Yamamoto‐Kinami, Yoshiyuki Tsuchiya, Tomomi Kanazawa, Yuki Hoshino, Keiichi Matsuda, Toshihiro Ichijo

**Affiliations:** ^1^ Graduate School of Veterinary Science Iwate University Morioka Iwate Japan; ^2^ Farm Animal Clinical Skills and Disease Control Center Iwate University Morioka Iwate Japan; ^3^ Cooperative Department of Veterinary Medicine, Faculty of Agriculture Iwate University Morioka Iwate Japan; ^4^ Miyagi Prefectural Federation of Agricultural Mutual Aid Association Kurokawa‐gun Miyagi Japan

**Keywords:** amino acids, calf, hepatic portal, liver, pre‐ruminant

## Abstract

Sufficient amino acids (AAs) supply is crucial in growing animals to maintain the rapid skeletal muscle protein synthesis and healthy growth. Liver is known to be the major organ that plays a central role in AA metabolism. Seeing as few studies have been made to investigate the dynamic changes of postprandial AAs over a short time interval before and after the liver, a first attempt was made to investigate the changes in postprandial free AA levels over eight time points with short interval in plasma, collected simultaneously from the hepatic portal, hepatic, and jugular veins, to better understand the intrahepatic, pre‐ and post‐hepatic AA metabolisms. AAs absorption and uptake by liver occurred soon after feeding and most of the AAs peaked at 30 min postprandial. Two postprandial peaks of the plasma total free AAs, essential AAs (EAAs), and non‐essential AAs were observed in hepatic portal and hepatic veins, indicating that two phases of digestion and absorption of AAs may occur in the gastrointestinal tract of the pre‐ruminant calves. Individual free AAs showed three main AA transition profiles over time. The total EAA concentration at 240 min postprandial was significantly higher (*p* < 0.05) in the HPV than in the JV.

## 1 Introduction

Amino acids (AAs) are generally defined as organic compounds consisting of both amine (‐NH_2_) and carboxyl (‐COOH) groups, and an organic R group (or side chain) that is unique to each AA (Sanz et al. 2019). AAs can be utilized for both glucose and lipids syntheses. However, the reverse process, i.e., producing AAs from either glucose or lipids, is not possible without other AAs acting as nitrogen donors (Dioguardi 2011). The free AAs absorbed from the gut enter the liver, which is an important organ for AA metabolism, via the hepatic portal vein (HPV), and are then transferred to various organs throughout the body (Wray‐Cahen et al. 1998).

Sufficient AAs supply is required in growing animals to maintain the rapid skeletal muscle protein synthesis and healthy growth (McDonald et al. 2010; Zheng et al. 2019), and to enhance lifetime productivity. A number of studies have been performed to investigate AA metabolism in calves (Houlier et al. 1991; Koeln et al. 1993). Catheterization has been a useful tool for repeated blood sampling from portal and hepatic veins (HVs) in long‐term studies. Houlier et al. applied catheter implantation in pre‐weaning calves for blood sampling from the portal and HVs, and the mesenteric artery for measurement of AAs in whole blood and plasma and revealed that AAs are mainly transported to the liver in the plasma (Houlier et al. 1991). In addition, Koeln et al. surgically implanted cannulas in the abdominal aorta, a distal mesenteric vein, the portal vein, and a HV for determination of AAs exchange across the gastrointestinal tract and liver, reporting that the net negative flux of free AAs across the liver during feed deprivation was greater than the flux across the gastrointestinal tract (Koeln et al. 1993). Mohamed et al. implanted catheters into the portal and HVs of mature cattle non‐surgically for blood sampling at 60 min before feeding and 180 min after feeding, together with sampling of jugular blood to investigate the postprandial changes in ammonia nitrogen, urea nitrogen, glucose, acetate, hydroxybutyrate, and lactate concentrations in the blood (Mohamed et al. 2002). Nevertheless, above‐mentioned studies monitored plasma AAs changes over time with 1 h or longer intervals. Whereas, on the basis of experience we assumed that absorption of dietary AAs by intestine, and AAs uptake by the liver may occur in a more rapid manner and show a more active movement during the several h immediate after ingestion. Therefore, we collected blood samples simultaneously from the hepatic portal, hepatic and jugular veins (JVs) over shorter intervals, at 30‐min intervals during the first 120 min, followed by 60‐min intervals until 240 min, and then at 360 min after ingestion in healthy pre‐ruminant calves, to make a close observation of the dynamic changes in postprandial free AA concentrations in the plasma during the several h immediate after feeding. Blood samples were collected from three different blood vessels, i.e., the hepatic portal, hepatic and JVs for the pre‐, intra, and post hepatic AA levels analysis, in order to investigate the AA metabolism in the liver in a more accurate manner.

## 2 Materials and Methods

All animal study procedures and protocols were approved by the Animal Research Committee and followed the guidelines for Animal Experiments at Iwate University, Iwate Prefecture, Japan (No. A201944).

### 2.1 Animals, Management, and Treatments

The study was conducted at the Livestock Clinic Block of the Iwate University Animal Hospital, Iwate Prefecture, Japan. Nine healthy pre‐weaning Holstein male calves purchased from commercial dairy farms in Iwate Prefecture, Japan, were enrolled in the study in two batches. The first batch of five calves were studied from December 2021 to February 2022, and the additional four calves were tested from December 2022 to February 2023. The calves were acclimated and housed in calf hunches inside the Livestock Clinic Block for 2 weeks after arrival. Milk replacer and calf starter were fed twice daily (9:00 a.m. and 4:00 p.m.), with ad libitum water and roughage offered at all times during acclimation. Body weight (BW) was measured and recorded once a week.

On the day of the study, milk replacer was only fed in the morning, without supply of calf starters and roughage. Milk replacer (500 g) was dissolved in 4 L of warm water (1000 g/8 L/day) and administered orally using a feeding bottle. From 4:00 p.m. on the day before the study, all calf starter and roughage were removed, and only 2 L of drinking water was provided. This feeding protocol aimed to minimize the potential impact of individual differences in the intake of roughage and calf starter prior to the study on the results. The nutrient and AA composition of the milk replacer is presented in Table S1. The calves (4.2 ± 1.4 weeks of age; 50.2 ± 8.1 kg of BW) were non‐surgically implanted with catheters in the HPV and in the HV with the guidance of ultrasound imaging in accordance with Nakajima et al. 2002, 2 h prior to the initiation of the study (Nakajima et al. 2002). Preliminary trials were performed and confirmed that this procedure had no impact on milk replacer intake or the safety of the calves. The catheters were washed with heparin sodium injection after the implantation and after each blood sampling during the study, respectively, to prevent blood clotting.

The calves remained healthy throughout the study and no visual signs of infection or inflammation were observed from results of the blood test and blood chemistry tests. There was failure of blood sampling from the HPV of one calf, thus the data for the HPV are the means of eight measurements.

### 2.2 Sampling and Laboratory Analyses

A series of eight blood samples were taken from the left JV, HPV, and HV with the aforementioned implanted catheters from each calf. The first sample was drawn 15 min before the morning diet, followed by the other samplings at 30, 60, 90, 120, 180, 240, and 360 min after feeding.

For AA analysis, 7 mL of blood was collected in EDTA‐2Na‐supplemented vacuum blood collection tubes, cooled down to 4°C immediately and centrifuged at 1560*g* for 10 min. Plasma was removed and frozen at −80°C until analysis. The general blood test was performed immediately after each sampling, with 2 mL blood samples collected in EDTA‐2Na‐supplemented vacuum blood collection tubes. For blood biochemical testing, another 7 mL blood was collected in a plain vacuum blood collection tube, kept at 37°C for 15 min, followed by centrifugation at 1560*g* for 15 min. Serum was collected and rapidly frozen and stored at −80°C until analysis.

The analysis of the free AA concentrations in the plasma samples was outsourced to a commercial testing laboratory (Obihiro Clinical Testing Laboratory, Hokkaido, Japan), and analyzed by LC/MS instrumentation (Matsumoto et al. 2014).

### 2.3 Statistical Analysis

Data was statistically analyzed using EZR Version 1.54. The data on plasma AA concentrations are presented as means ± standard deviation of the mean (means ± SD). Determination of significance of changes along all‐time points for the same blood vessel was performed by univariate type III repeated‐measures ANOVA, which accounts for the repeated measurement within each subject and assesses the overall effect of time on the measured variable, additionally applied with Bonferroni correction to adjust the significance level. Multiple comparisons among blood vessel at the same time points were conducted using the Tukey–Kramer method, considering differences in sample sizes. The significance level was set at *p* < 0.05.

## 3 Results

### 3.1 Plasma Free AAs (PFAAs) Concentration After Meal Ingestion

**FIGURE 1:**
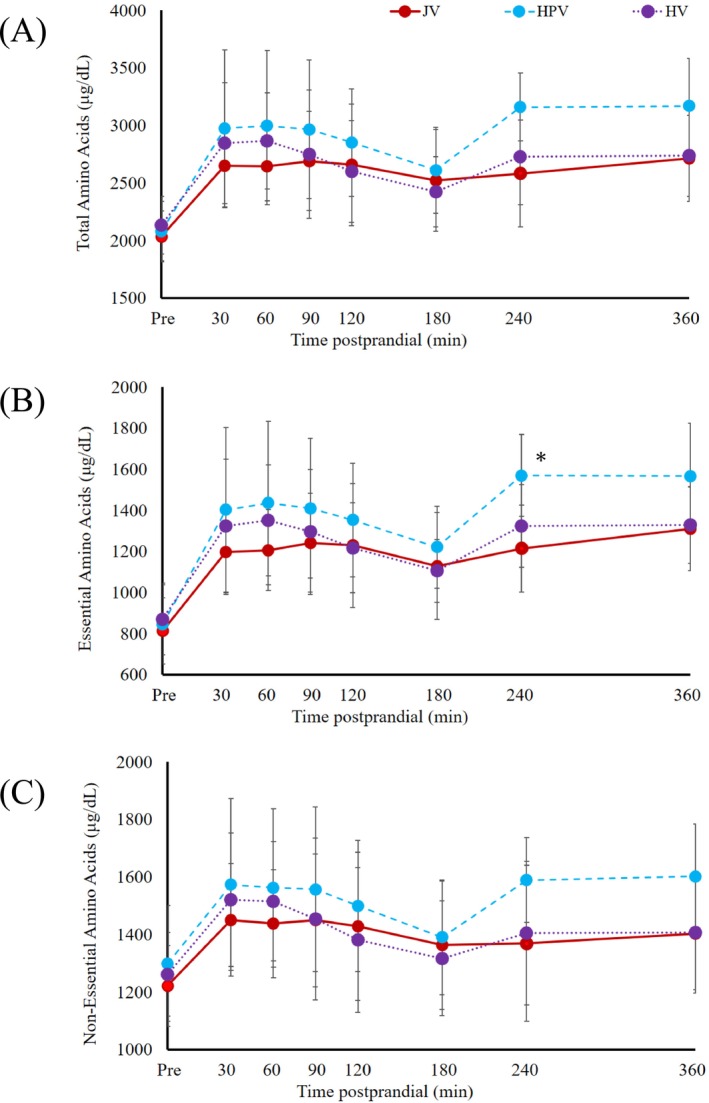
Total free amino acid (A), essential amino acid (B), non‐essential amino acid (C) levels in blood plasma (μg/dL; means ± SD) collected simultaneously from the hepatic portal vein (HPV; blue circle), hepatic vein (HV; purple circle), and jugular vein (JV; red circle) in pre‐weaning calves after a single meal. *Asterisks indicate statistically significant difference (*p < 0.05) between the HPV and JV values at a given sample time.

The concentrations of the plasma total free AAs (TAAs) concentration, essential AAs (EAAs; arginine, histidine, isoleucine, leucine, lysine, methionine, phenylalanine, threonine, tryptophan, and valine) concentration, and non‐EAAs (NEAAs; alanine, aspartic acid, cysteine, glutamic acid, glutamine, glycine, hydroxyproline, proline, serine, and tyrosine) in all three blood vessels increased rapidly during the first 30 min, remained until 90 min, and decreased gradually between 90 and 180 min. A similar two peaks profile for TAA, EAA, and NEAA was observed in the HPV and HV samples (Figure 1A–C), where the first peak occurred at 30 min or 60 min, and the second peak at 240 min after ingestion.

### 3.2 Individual Free AAs Uptake Profiles After Ingestion

**FIGURE 2:**
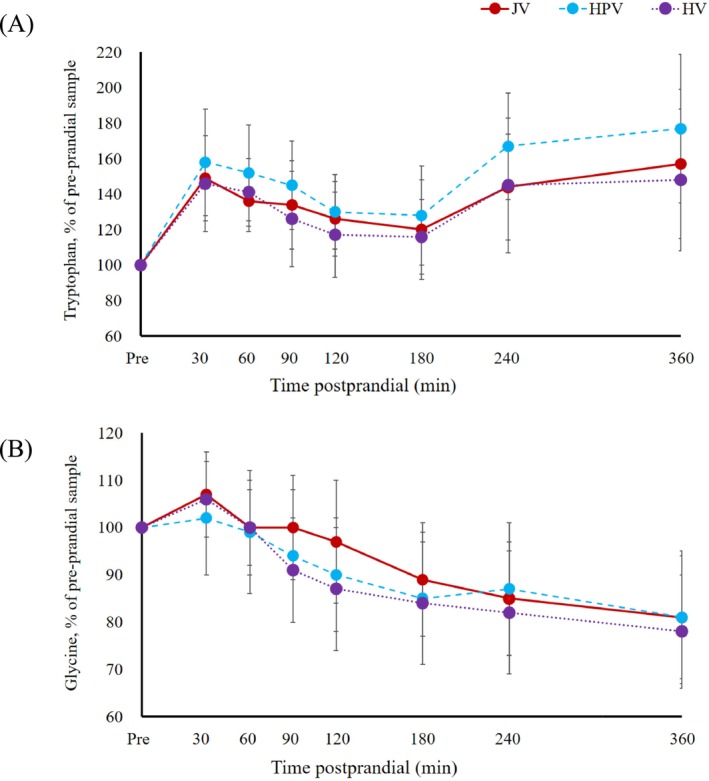
Plasma tryptophan (A) and plasma glycine (B) levels (% of pre‐prandial samples; means SD) in blood samples collected simultaneously from the hepatic portal vein (HPV; blue circle), hepatic vein (HV; purple circle triangle), and jugular vein (JV; red circle) in pre‐weaning calves after a single meal. Statistically significant difference was not observed between blood vessels in all AA uptake types at all sampling time points.

The postprandial concentrations of individual free AAs in the plasma varied markedly from one sampling time to another. Among these, tryptophan and glycine showed distinct transition patterns as representative AAs (Figure 2A,B). Postprandial plasma tryptophan level demonstrated a biphasic response. There was a rapid initial increase in the concentration postprandial, peaking at 149% of the pre‐prandial level in the JV at 30 min (*p* < 0.05), at 158% in the HPV, and at 146% in the HV (*p* < 0.05) at the same time. Following this peak, there was a gradual decline, but a second rise was observed, reaching 157% in the JV (*p* < 0.05), 177% in the HPV (*p* < 0.05), and 148% in the HV at 360 min, respectively. In contrast, plasma glycine level showed a steady decline postprandial. In the JV, postprandial plasma glycine level decreased from 107% of the pre‐prandial level at 30 min to 81% at 360 min. Similarly, the concentration in HPV fell from 102% to 81%, and in HV, it decreased from 106% to 78% (*p* < 0.05) over the same time period. Except for glycine and hydroxyproline showing close to peak concentrations at feeding and decreasing constantly between 30 and 360 min after feeding, the concentrations of all other free AAs in the plasma were always above their baseline levels at all the sampling time points within 360 min after feeding (Tables S2 and S3).

### 3.3 Differences Between Blood Vessels

Significant differences between blood vessel samples were not observed for most of the time points, except at 240 and 360 min where plasma concentrations of TAA and EAA were significantly higher (*p* < 0.05) in the HPV sample than both the HV and JV samples (Figure 1A,B). The change over time of most of the individual PFAA concentrations (*p* < 0.05) was observed more often in the HPV samples than in the HV and JV samples. Several EAA (isoleucine, leucine, phenylalanine, and valine) and NEAA (proline and serine) concentrations in the plasma showed significant differences (*p* < 0.05) between the sampled blood vessels at certain time points, but the concentrations of other AAs did not differ between the sampling sites. At 360 min postprandial, serine concentrations in the HPV were significantly higher (*p* < 0.05) than those in the JV. Similarly, at 240 min postprandial, proline, valine, isoleucine, and leucine levels were significantly higher (*p* < 0.05) in the HPV compared with the JV. Additionally, at 240 min postprandial, phenylalanine concentrations were significantly higher (*p* < 0.05) in both the HPV and HV than in the JV (Tables S2 and S3). Consequently, the total EAA concentration at 240 min postprandial was significantly higher (*p* < 0.05) in the HPV than in the JV (Figure 1B). Notably, glutamic acid uptake profile displayed unique dynamic changes among the three sampling sites as compared with other AAs, where the concentrations in the HV consistently maintained higher glutamic acid levels as compared with both the HPV and JV at all measured times postprandial.

## 4 Discussion

In this study, a rapid rise in plasma TAA, EAA, and NEAA concentrations at the first 30 min postprandial was observed in all blood vessels. The PFAAs peaked at 30–60 min after feeding, followed by a decrease and an increase again to a second peak around 240 min, and did not return to baseline levels by the end of the study. The results supported our assumption that absorption and metabolism of dietary AAs by intestine and liver occurred rapidly and actively during the first few hours after ingestion. Diphasic AA curves observed in this study suggest that two phases of digestion and absorption of AAs may occur in the gastrointestinal tract of the pre‐ruminant calves. This biphasic pattern highlights the rapid and dynamic metabolic processes in pre‐ruminants, which are distinct from those in mature ruminants.

AA metabolism differs significantly between pre‐ruminant and mature ruminant animals due to the developmental stage of the digestive system. In mature ruminants, such as cattle and goats, rumen fermentation plays a dominant role in altering the profile of dietary AAs entering the small intestine. Microbial protein synthesized in the rumen becomes the primary source of AAs absorbed post‐ruminally (Koeln et al. 1993; Li et al. 2022). This process leads to a more gradual and continuous release of AAs into the bloodstream compared with pre‐ruminants.

At the same time, the biphasic pattern observed in the calves may also be partially explained by curd formation (milk coagulation) in the abomasum, as reported by Miyazaki et al. (2009). The study demonstrated that curd formation occurs between 60 and 120 min after ingestion of milk replacer. A more recent article from the same group (Miyazaki et al. 2017) also revealed a slight reduction in the size of the abomasal curd was observed from 2 h after ingestion, which may play a role in the slow release of a protein source (e.g., casein) for efficient AAs or peptides absorption in the small intestine. Additionally, curd formation in the abomasum has been shown to decelerate the duodenal flow of fat and nitrogen from milk replacer (Petit et al. 1987), further supporting its role in the observed biphasic AA absorption.

In addition, there are also studies showing that pancreatic juice secretion induced two postprandial AA peaks (McCormick and Stewart 1966), where the first peak was a result of cephalic stimulation driven by the vagal cholinergic pathway, and the second peak was a result of the intestinal phase coinciding with elevated flow of digesta through the small intestine (Pierzynowski et al. 1992). For those AAs that showed two peaks, the second peak may reflect digestion and absorption of the slow releases AAs from the abomasal curd, in addition to the AAs from protein digestion promoted by the second peak secretion of pancreatic juice.

The results agreed with an earlier report that individual AAs are not absorbed with equal efficiency (Webb 1990). The difference in free AA uptake trends may be explained by different mechanisms involved in the AA's absorption, where the absorption of AAs can occur through either passive (facilitated or simple diffusion) or active (Na+ or H+ co‐transporters) pathways (Frenhani and Burini 1999). AAs absorption by passive transport activities is concentration‐dependent (Boge et al. 2002; Matthews 2000), whereas active AAs transport activities involve a variety of transporters (Bröer and Fairweather 2018). Distinct AA transporters have different affinities for specific AAs. Although transport of individual AAs generally takes place through more than one transporter, the number of transporters will also influence the pattern of a particular AA's uptake from the small intestine. Tryptophan, which is an EAA, is essential for protein synthesis, muscle development, and overall growth in rapidly growing pre‐weaned calves. It also plays a key role in synthesizing kynurenine, which has immunomodulatory properties (Wu et al. 2011). Tryptophan uptake pattern suggests a complex regulation of tryptophan absorption and metabolism, potentially involving delayed digestive processes or secondary metabolic pathways. On the other hand, glycine, which is a NEAA that plays several critical roles in the growth and development of pre‐weaned calves, such as protein synthesis, immune function, energy production, and gut health (Wang et al. 2013). The consistent decrease indicated a continuous utilization of glycine in metabolic processes, such as protein synthesis and conversion into other metabolites.

In spite of the patterns of the AA uptake dynamics, all individual plasma‐free AA, with the exception of glycine and hydroxyproline, elevated with double peaks within the 360 min after ingestion and did not return to baseline levels by the end of the study. Concentrations of plasma glycine and hydroxyproline started decreasing from 30 min after ingestion, remained low between 30 and 360 min in all three blood vessels, and were lower than baseline levels at 120 min and thereafter. The observation of a decrease in plasma concentrations of both glycine and hydroxyproline in this study was consistent with an earlier study (Rony et al. 1975). In addition, plasma glycine levels lower than baseline at 4 h post‐feeding in pre‐weaning calves (Ghaffari et al. 2017), and decreased plasma glycine concentrations at 30 min after ingestion in growing pigs (Li et al. 2019) were also reported. Synthesis of glycine takes place in the body from serine, threonine, choline, and hydroxyproline through inter‐organ metabolism involving primarily the liver and kidneys. Under normal feeding conditions, glycine is not sufficiently synthesized to keep up with its utilization in (1) the glycine cleavage system for formation of ammonia and CO_2_; (2) the biosynthesis of glutathione, heme, creatine, nucleic acids, and uric acid; and (3) the conjugation with bile acids to increase their solubility, and to be secreted into the small intestine in response to dietary fat (Wang et al. 2013). On the other hand, hydroxyproline has been traditionally considered to have little nutritional importance in animals but is presently recognized as a substrate for the synthesis of glycine, pyruvate, and glucose, which are particularly important for neonates and ruminants (Wu et al. 2011). The findings of a negative balance in both the postprandial glycine and hydroxyproline levels indicated inadequate supply of the two AAs through the diet.

With the exceptions of glycine and hydroxyproline, all individual free AAs remained above baseline levels at the end of the study, indicating that more than 360 min is required for the completion of the PFAAs cycle after feeding in pre‐weaning calves. There is a report that the PFAAs postprandial cycle is fully completed 13 h after ingestion in young dairy calves (Rony et al. 1975).

Significant differences between blood vessel samples were not observed for most of the time points. Except for aspartic acid, cysteine, glutamic acid and glutamine, most of the free AAs showed similar curves in all three blood vessels throughout the study. The elevated level of serine, proline, valine, isoleucine, and leucine in the HPV beyond 240 min postprandial is particularly noteworthy. Notably, valine, leucine, and isoleucine, which are classified as branched‐chain AAs (BCAAs), may require an extended period for absorption from the gastrointestinal tract, suggesting a delayed uptake mechanism in pre‐ruminant calves (Tables S2 and S3). Additionally, although statistically significant difference was not observed between blood vessels, the plasma glutamic acid concentration in the HV was higher than those in the HPV and JV during this study after feeding. Dietary glutamic acid is metabolized to a great extent in the gastrointestinal tract, and Reeds et al. (1996) revealed that almost no enteral glutamic acid is observed in portal venous blood. Rather, the high level of plasma glutamic acid in the HV is considered to be the result of hepatic metabolism and transamination of AAs and the detoxification of ammonia. The “glutamate family” of AAs (arginine, ornithine, proline, histidine, and glutamine) need to be converted to glutamate for their metabolic disposal, where these AAs comprised about 31% of the dietary AA intake in the present study. Besides, most AAs are partially oxidized in the liver, with the bulk of their carbon skeleton being converted to glucose, and the nitrogen being converted to urea and to a lesser extent glutamine (John 2000).

In conclusion, our study demonstrated that AA absorption actively occurred immediately after feeding in pre‐weaning calves. As a result of the short‐interval blood sampling and analysis within the first few hours after feeding, biphasic plasma AA curves were observed, indicating that two phases of digestion and absorption of AAs may occur in the gastrointestinal tract of the pre‐ruminant calves. Although significant differences between blood vessel samples was not observed for most of the time point, the study results provide insights into the intrahepatic, pre‐ and post‐prandial metabolisms of PFAAs in calves. These findings provide a foundation for future studies aimed at optimizing nutritional strategies and therapeutic interventions, particularly under varying physiological and pathological conditions. For instance, the observed biphasic plasma AA curves suggest that tailored feeding regimens could improve AA utilization efficiency in pre‐weaning calves. Additionally, this study provides a baseline for investigating AA absorption and metabolism in calves suffering from conditions such as diarrhea or metabolic disorders, paving the way for developing targeted dietary or therapeutic approaches to enhance recovery and growth.

## Conflicts of Interest

The authors declare no conflicts of interest.

## Supporting information


**Table S1.** Chemical and amino acid composition of the diet TDN; total digestable nutrients.

**Table S2.** Individual free essential amino acid (EAA) levels in hepatic portal, hepatic, and jugular plasma of pre‐weaning calves collected simultaneously pre‐ and postprandial (μg/dL; means ± SD). Values are means ± SD. HPV, hepatic portal vein; HV, hepatic vein; JV, jugular vein. Asterisks indicate a statistically significant difference (*p < 0.05) between the HPV and JV values at a given sample time. Black squares indicate statistically significant difference (^■^ *p* < 0.05) between the HPV and HV values at a given sample time. Values from the same vessel followed by the same letter do not differ significantly (*p* < 0.05).

**Table S3.** Individual free non‐essential amino acid (NEAA) levels in hepatic portal, hepatic, and jugular plasma of pre‐weaning calves collected simultaneously pre‐ and postprandial (μg/dL; means ± SD). Values are means ± SD. HPV, hepatic portal vein; HV, hepatic vein; JV, jugular vein. Asterisks indicate a statistically significant difference (*p < 0.05) between the HPV and JV values at a given sample time. Values from the same vessel followed by the same letter do not differ significantly (*p* < 0.05).
